# A Study of Professional Awareness Using Immersive Virtual Reality: The Responses of General Practitioners to Child Safeguarding Concerns

**DOI:** 10.3389/frobt.2018.00080

**Published:** 2018-07-12

**Authors:** Xueni Pan, Tara Collingwoode-Williams, Angus Antley, Harry Brenton, Benjamin Congdon, Olivia Drewett, Marco F. P. Gillies, David Swapp, Pascoe Pleasence, Caroline Fertleman, Sylvie Delacroix

**Affiliations:** ^1^Department of Computing, Goldsmiths, University of London, London, United Kingdom; ^2^Department of Computer Science, University College London, London, United Kingdom; ^3^BespokeVR Ltd, London, United Kingdom; ^4^Medical School, University College London, London, United Kingdom; ^5^Faculty of Laws, University College London, London, United Kingdom; ^6^Great Ormond Street Institute of Child Health, University College London, London, United Kingdom; ^7^Whittington Health, London, United Kingdom; ^8^Birmingham Law School, University of Birmingham, Birmingham, United Kingdom

**Keywords:** immersive virtual reality, virtual patient, medical training, professional awareness, child safeguarding, expertise, cognitive load, naturalistic decision making

## Abstract

The art of picking up signs that a child may be suffering from abuse at home is one of those skills that cannot easily be taught, given its dependence on a range of non-cognitive abilities. It is also difficult to study, given the number of factors that may interfere with this skill in a real-life, professional setting. An immersive virtual reality environment provides a way round these difficulties. In this study, we recruited 64 general practitioners (GPs), with different levels of experience. Would this level of experience have any impact on general practitioners' ability to pick up child-safeguarding concerns? Would more experienced GPs find it easier to pick up subtle (rather than obvious) signs of child-safeguarding concerns? Our main measurement was the quality of the note left by the GP at the end of the virtual consultation: we had a panel of 10 (all experienced in safeguarding) rate the note according to the extent to which they were able to identify and take the necessary steps required in relation to the child safeguarding concerns. While the level of professional experience was not shown to make any difference to a GP's ability to pick up those concerns, the parent's level of aggressive behavior toward the child did. We also manipulated the level of cognitive load (reflected in a complex presentation of the patient's medical condition): while cognitive load did have some impact upon GPs in the “obvious cue” condition (parent behaving particularly aggressively), this effect fell short of significance. Furthermore, our results also suggest that GPs who are less stressed, less neurotic, more agreeable and extroverted tend to be better at raising potential child abuse issues in their notes. These results not only point at the considerable potential of virtual reality as a training tool, they also highlight fruitful avenues for further research, as well as potential strategies to support GP's in their dealing with highly sensitive, emotionally charged situations.

## Introduction

Aside from having to grasp an ever-growing body of medical knowledge, today's general practitioners (GPs) need to be equipped with a wide set of practical and social skills. While some of those skills can be taught pretty straightforwardly, others are harder to inculcate without the benefit of experience and role models. The ability to pick up signs that a child may be suffering from abuse at home is one of those skills that cannot easily be taught. The clearly non-cognitive underpinnings of this skill made it an ideal focus point for this study, which, at a more abstract level, is driven by an endeavor to develop a better understanding of the non-cognitive aspects of professional expertise. To become a professional indeed requires a process of habituation, whereby one comes to internalize “the way things are done.” Aside from its cognitive elements, the latter typically encompasses a mix of intuitive understandings and both reflective and unreflective habits. These non-cognitive, deeply internalized aspects of expertise can be what distinguishes the merely competent from the truly brilliant: just as experienced firemen seem able to sense when to evacuate a building that is about to collapse, some healthcare providers seem able to sense when something is amiss with a child even in the absence of any concerns expressed by the child and/ or her carer.

In the UK, all GPs are entrusted with the responsibility to identify potential child abuse issues and keep record of any concerns they may have (General medical council, 2012), given their position as primary point of contact for families with healthcare concerns. Beyond the specific child protection training provided in the context of continuous education - all GPs have to obtain level 3 safeguarding competency (Royal College of Paediatrics Child Health, 2014), undergraduate education about child protection has only been included in the medical school curriculum relatively recently. It is mentioned in the American literature as early as 1996 (Dorsey et al., 1996), but, as an example, in UCL Medical School it was only formally included from 2005. A variety of teaching styles is used, often mirroring postgraduate training, but delivered appropriate for the learners' level of knowledge, exposure and experience (Hann and Fertleman, 2016). A qualitative study at UCL of medical students' experience of child protection teaching by Yiannis Ioaunnou concluded “*these students have placed great emphasis on emotional aspects of the subject. They have commented on their uncertainty of their own role in these situations and concern about managing emotions that might be experienced*” (Ioannou, 2008).

Emphasis on the impact of emotions on the processes that are constitutive of morally-loaded judgments is far from new. The dominant, dual-process theory highlights the interaction between cognitive processes on one hand, and emotional and intuitive processes on the other. Cognitive load manipulation experiments have long been relied on to throw light on these interaction modalities. They have largely contributed to corroborating the now widely influential “dual system” theory (Kahneman, 2011), which highlights a dichotomy between two different ways in which we may apprehend a given situation: while System 1 produces fast, instinctive and emotional answers, System 2 stands for slower, deliberative modes of thought. The latter are meant to supervise System 1's fast, emotional and/or intuitive answers. When cognitive load disrupts System 2's supervising role, intuitions and emotions are given free(er) rein. This can prove problematic and lead to an increase in erroneous judgments (Gilbert, 1989; Menaker et al., 2006; Pawar et al., 2017) particularly so when those judgments proceed from simplifying heuristics rather than a skill learned from experience.

The “naturalistic decision making” tradition (NDM), which owes its name to an endeavor to study how people “actually” make decisions under conditions (like high stakes or team dynamics) that are not easily replicated in the laboratory (Klein, 2008; Zsambok and Klein, 2014) has focused on the latter, skilled type of intuition. Initially developed from an attempt to analyze the way fireground commanders make decisions under conditions of uncertainty and time pressure (which hamper System 2's ability to systematically evaluate a set of options), naturalistic decision making has since studied many professions-specific examples of what it refers to as “skilled” intuitions. Acquired through extensive experience in an environment that allows for systematic, constructive feedback, those skilled intuitions are contrasted to those that arise from quick, simplifying heuristics that are never put to the test. (Crandall and Getchell-Reiter, 1993) for instance studied the intuitions that allow nurses in a neonatal intensive care unit to detect life-threatening infections even before blood tests came back positive. These intuitions draw upon tacit, rather than explicit knowledge: the nurses' remarkable ability was acquired through extensive experience, rather than any formalized training based on a set of rules or principles. Henceforth we will refer to the above as the “skilled intuition effect.”

While the Naturalistic Decision Making tradition is widely acknowledged as having the potential to contribute in a substantial way to our understanding of the factors that impact upon professional judgment, it is often criticized due to its having to study professional judgment “*in situ*,” with the lack of controllability this entails. In this respect, reliance on immersive virtual reality to study ecologically valid professional judgments -albeit in a controlled environment- has great potential as an added “tool” at the disposal of all those seeking to gain a better understanding of the factors that impact upon professional judgment (within or without the Naturalistic Decision Making tradition), since it allows for those factors to be controlled and replicated with a high degree of precision.

Using virtual humans in the field of medical training is not new. For instance, early work by Johnsen et al. (2007) showed a significant correlation between medical students' performance with a virtual and a human patient, and (Raij et al., 2007) showed that medical students were able to elicit the same information from the real and virtual human (although showing less interest and poorer attitude toward the latter). More recently, virtual patients have been used in training for mental health assessment (Foster et al., 2015; Washburn et al., 2016), empathetic communication (Kleinsmith et al., 2015), and identifying gender bias in diagnosis (Rivera-gutierrez et al., 2014). In these studies, typically, human participants interacted with virtual patients via text or voice, and the virtual patients were animated and programmed to react toward the participants in a realistic way. For a summary of different types of virtual patients see (Talbot et al., 2012; Kononowicz et al., 2015).

Our approach emphasizes the ecological validity of the GP-virtual patient interaction: our use of immersive virtual reality allows the participants to interact with 3D, human-sized and realistically animated virtual patients. Participants are able to interact with these virtual characters in the most natural way using their voice and gestures, and the virtual characters respond appropriately, using a wizard-of-oz method where an experimenter selects the reaction from a command window. Similar approaches have been used in previous work including therapy for social anxiety (Pan et al., 2012), study of bystander reactions to a violent incident (Slater et al., 2013), and more recently training for GPs to resist the unreasonable demand to prescribe antibiotics (Pan et al., 2016).

This study was designed to test whether the level of professional experience—as well as cognitive load—have any impact upon a GP's ability to correctly identify (and devise some strategy to address) child safeguarding concerns. As one of the advantages of using virtual reality consists in its allowing for the accurate control of various factors in the GP-patient interaction, in this work we controlled the level of child abuse cues. The cues were made to be more obvious or more subtle by manipulating only the level of aggressiveness shown by the parent toward his son during the consultation. The behavior of the son remained the same in both conditions (obvious v. subtle cues). We wanted to test whether the more obvious cues from a “virtual parent” would make it easier for the GPs to identify child-safeguarding concerns and take appropriate actions. The design of the scenario itself (based on a real-life case study) was chosen because its most important child-safeguarding cues predominantly require perceptual awareness rather than cognitive engagement on the part of the GP. The medical condition of the parent, which was presented in a more or less complex set of letters (high v. low cognitive load conditions) from two cardiologists, was also based on a real-life case study and adapted with specialist advice to retain maximum plausibility.

Overall our research questions are as follows:

**Research question 1:** Does the degree of professional experience impact upon a GP's ability to identify and act upon child safeguarding concerns effectively?
*Hypothesis 1*: Highly experienced GPs will be more likely to pick up child-safeguarding concerns and act upon it more effectively (skilled intuition effect, discussed above).
*Hypothesis 2*: Highly experienced GPs will be better able to pick up subtle (as opposed to obvious) signs of child-safeguarding concerns than their inexperienced counterparts.
**Research question 2**: Does cognitive load affect a GP's ability to pick up signs of child-safeguarding concerns and act upon it effectively?
*Hypothesis 3*: Cognitive load will affect all GP's ability to pick up signs of child-safeguarding concerns and act upon it effectively.
*Hypothesis 4*: The impact of cognitive load will be greater on less experienced GPs.

## Materials and methods

### Ethics statement

The study was approved by and carried out in accordance with the regulations of the Research Ethics Committee of UCL. Participants gave written informed consent on a form devised for this purpose that had been approved by the said Research Ethics Committee.

### Scenario details

Video link: http://www.panxueni.com/gpcave

Our previous work used the HMD system where participants (i.e., GPs or trainee GPs) were fully immersed in the virtual interactions with a head-mounted display (Pan et al., 2016). Many participants commented that they found not having access to a computer made the experience less real, as they always relied on a computer during their real-life consultations. This presented us with a challenge: as the HMD systems block the real world completely, it is not possible to be immersed in VR while having access to a real computer at the same time. It is also not possible to simulate a virtual computer inside the HMD because the HMD's display resolution remains too low. In order for the participants to have access to a real computer while being immersed in the virtual environment, a CAVE-like system was used.

**Figure 1:**
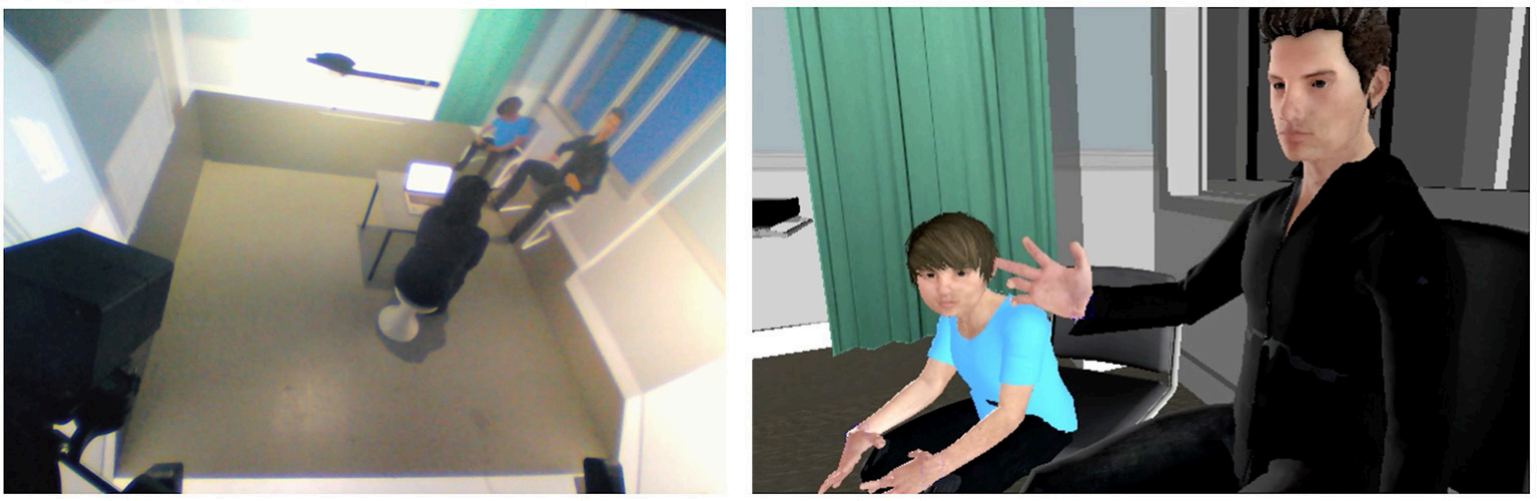
Medical Doctor interacting with virtual patients in a CAVE-like system.

A virtual consultation was created in Autodesk Maya using some assets downloaded from the Unity assets store. The layout is modeled based upon photographs we took of a GPs consultation room in the United Kingdom, with a few chairs, a medical bed behind a curtain, some medical information posters hanging on the wall, and a hand sterilizer next to the door. As shown in Figure 1, the configuration of the room is carefully designed to reflect NHS guidelines where the patients' seating area is not blocking the doctor's access to the exit door. The participant, who is either a GP or a trainee GP, also has a real desktop and laptop in front of them just like they would in a real consultation. On the laptop, they have access to notes related to their next patient, Mr. Christopher (Chris) Truman, including basic information (date of birth, gender, NHS number) and two expert consultation letters. The two letters both indicate that Mr. Christopher Truman needs an operation for his aortic stenosis but give different recommendations: one suggesting trans-catheter aortic valve implantation (TAVI) and the other one an open-heart surgery. There were two sets of these letters as part of the experimental condition (see Supplemental Material: consultation letters). On the laptop, the participant also has an area where they are free to type in their notes. Everything is supported by a very unsophisticated web-browser interface and participants were given time to familiarize themselves with it and to study the notes before their consultation.

As the VR scenario starts, the participant finds themselves sitting on their own in the consultation room, with the door ajar. A man approaches the room and asks politely if the participant is expecting to see a “Christopher Truman,” and once confirmed, he apologizes that he has to bring his 6-year-old son with him. He then enters the room with his son, Tom, following him.

Chris sits down and becomes very upset with Tom when he realizes that, instead of sitting on the chair properly, Tom is staring at the chair. At this point Chris appears to have lost his patience. He aggressively gestures toward Tom to sit down (without touching Tom's body), and Tom flinches. Chris then apologizes for being bad tempered blaming his poor health, and the consultation carries on (see Supplemental Material: Video).

Chris explains that he needs to understand the options he has in order to make a quick decision to secure the surgery slot he has been offered. Typically, the participant and Chris would spend between 3 and 5 min discussing his options: open-heart surgery which is more risky but a permanent solution, or TAVI which is a less risky procedure but only a temporary solution. Toward the end of their discussion, Chris asks the participant to clarify what is the worst that could happen if he went for the open-heart surgery and is shocked to discover that he could die.

While Chris is deep in his thoughts, Tom interrupts and says that he wants to go to the toilet. Chris ignores Tom. Tom repeats his demands with a raised voice which makes Chris very upset. He shouts at Tom and tells him to hold on as he is having an important conversation.

At this point Chris's phone rings. It is clearly a phone call he has been waiting for. He quickly picks it up with and walks out of the room while talking on the phone, leaving the door closed behind him.

The moment Chris walks out of the room Tom looks visibly more relaxed. The participant has the opportunity to ask questions, they are free to ask anything but typically they would ask questions such as “What's your name?” “How old are you?” “Do you have any brothers or sisters?” As and when prompted by the participant, Tom answers these questions with short replies. If the participant chooses to ask questions related to Chris and how things are at home, Tom looks down or nods.

The opportunity for the participant to interact with Tom lasts 1 min—until Chris re-enters the room. He apologizes for having to pick up the important phone call from his brother. At the same time, he informs the participant that he has made up his mind and will go for the open-heart surgery. He then leaves the room with Tom. The participant is then left to type up their consultation notes on the laptop.

### Design

The experiment has a 2 × 2 between group design with the two factors being: cognitive load (LOAD - two levels: high - H, low - L), and child abuse cue (CUE -two levels: obvious - O, subtle - S). In total, there are 4 conditions (HO, HS, LO, and LS). A power analysis was conducted for ANOVA assuming 80% power (α = 0.05). This analysis suggested that in order to detect a large effect (partial η^2^ = 0.14) with any of the cognitive load or child abuse cue factors (or an interactive effect), a total sample size of 52 would be needed. We recruited a total of 64 participants to take into account the fact that we did not know in advance the amplitude of the effect of our cognitive load manipulation.

In the high cognitive load version (HO and HS), the two letters were both very detailed and long and difficult to read; whilst in the low cognitive load version (LO and LS), they were both very brief and the key points were clearly highlighted with bullet points.

With the other factor, in the obvious cue version (HO, LO), the scenario was played out as descripted above; in the subtle cue version (HS, LS), the two points where Chris' became upset with Tom (after Chris sat down and realized Tom was still standing, and when Chris was interrupted by Tom who wanted to go to the bathroom), Chris behaved less aggressively (this manifests itself through his choice of words, tone of voice, and slightly less angry gestures -i.e., more gentle, less intimidating). In both conditions Tom behaved exactly the same. Our design and implementation for the child abuse cues were supervised by one of our co-authors (CF) who is also the senior co-author of “The Child Protection Practice Manual” (Hann and Fertleman, 2016). It is important to note that even in the subtle cue conditions, there are plenty of signs of child abuse: experts in this area would identify potential problems from the beginning when Tom walks in behind Chris as there is a clear, uncomfortable power dynamic, whereby Chris is dismissive of Tom. The GPs' concerns should be further confirmed when Chris ignores Tom's request to go to the toilet. There are also worrying signs when Chris talks about death in front of his son, and when Tom becomes visibly more relaxed as Chris leaves the room (most children would be more worried when a parent left them with a stranger).

Upon confirmation of their participation, participants were divided into two groups (GP, trainee GP) and within each group they were assigned to one of the four experimental conditions pseudo-randomly. This was to ensure that we had, as much as possible, an equal number of GPs and trainee GPs in each condition, and that the total number of participants in each condition was similar.

### Materials

The experiment was conducted in a CAVE-like virtual reality system at UCL. A detailed specification of the technical setup follows. The system conforms with the most common setup for CAVE systems, with three back-projected vertical screens (front, left and right, each 3 × 2.2 m) and a front-projected floor screen filling the enclosed space (3 × 3 m). The simulation was run on a workstation with nVidia K5000 graphics, delivering quadbuffer stereo to drive 4 Christie Mirage DLP projectors, each of which projected to one of the 4 screens (refresh rate 96 Hz). The display resolution was 1,400 × 1,050 for each of the vertical screens and 1,100 × 1, 050 for the floor screen. The graphics quality achieved is state-of-the-art; incrementally better performance can be achieved with the more recent graphics hardware (e.g., nVidia K6000 or AMD FirePro, or with higher resolution (4K) projectors). However, for this simulation the difference would not likely be noticeable. Resolution issues in CAVE setups typically manifest when the user stands close enough to the screens that the individual pixels can be seen. For this experiment, participants were seated over 1 m away from the nearest screen. Participants wore active stereo glasses (Volfoni ActivEyes Pro) to view the stereo imagery. Active stereo (as opposed to polarizing lenses) is the preferred technology for immersive VR system since it is better at eliminating cross-talk between the left and right eye images. In particular, we chose the Volfoni glasses due to their large lens size (50 × 37 mm) so that the frames do not encroach on the user's field of view. The position and orientation of the glasses was tracked by a 6-camera ART TrackPack system. This is an IR marker-based optical tracking system with 100 Hz update rate, with the recorded position/orientation being used to render the virtual scene from the wearer's viewpoint. ART TrackPack is widely used in CAVE setups due to its high accuracy over the full volume of the CAVE and the requirement for the user to wear only lightweight passive markers (attached to the glasses). The VR scenario was programmed in Unity3D and compiled with the MiddleVR middleware in order to run in the CAVE. Events were triggered by an experimenter through a control panel, which is set up on a separate desktop machine connected to the VR application machine via an internal network.

### Procedures

Sixty-four participants were recruited for the study. All were General Practitioners (GPs) or trainee GPs. They all visited the CAVE lab at UCL.

Before the study, participants read the information sheet which informed them that the study was designed to test whether virtual reality equipment can be used to simulate a realistic clinical consultation environment, and that they would be interacting with 3D avatars who are programmed to act as patients in a clinical setting. They then signed the consent form and completed a set of questionnaires, including demographics questions and other personality.

Participants were then guided to sit on a stool in front of a desk in the CAVE and to familiarize themselves with the interface. They were verbally informed that they would have a consultation with virtual patients, and that they should behave as they would during a real consultation. They were shown the laptop on the desk, which allowed them to read and type in notes. Other than the stool, desk, and laptop, which were real, everything else was computer generated 3D graphics. Participants were asked to inform the experimenter when they were ready. They were asked to put the shutter glasses on and the curtain behind them was closed.

At that point the participants witnessed the following 3D virtual scenario:

*Christopher shows up by the door and asks the Doctor if he is in the right room, and whether he can bring in his son Tom with him. The consultation then starts as described in “Scenario Details”. After Christopher and Tom leave the room, the screen goes dark and the participant completes the post-consultation notes on the (real) laptop provided*.

Afterwards the participants were asked to watch the video recording of their behavior during the virtual consultation and to provide a running commentary of their thoughts and feelings during the consultation. This commentary was also recorded. Finally, participants completed further questionnaires concerning their decisions during the consultation and the usefulness of Virtual Reality as a training tool. All participants were paid for their time (£20 store vouchers), as well as awarded with continuing professional development (CPD) points for completing 1-h training. At the end of the experiment all the participants received a certificate showing that they had undertaken an hour of level 3 safeguarding training.

### Response and explanatory variables

The main concern of this study is the extent to which the medical doctors are able to not only identify potential safeguarding issues, but also act effectively and timely. Hence our key measurement, in this quantitative analysis, focuses on the doctor notes left on the laptop immediately after the consultation.

In order to quantify our response variable, 10 raters were recruited to rate the post-consultation notes from all 64 participants independently. The raters were chosen from a wide variety of backgrounds, all experienced in safeguarding, and received formal teaching and training in this area. They were completely blind to the difficulty of the scenario cues or the level of complexity of the medical interview and had no demographic information about the participants. They had not been recruited to undertake the study themselves nor could they tell from the consultation notes who had written them.

Notes from all 64 participants were evaluated by those raters with a visual analog scale (VAS) score ranging from 0 (not notion of safeguarding) to 100 (fully demonstrates safeguarding concerns). We also collected some demographic data on the raters. They are salaried GPs, GP trainees, one GP lecturer, two pediatricians, and the two clinical medical students (both completed an intercalated BSc in pediatrics and child health). It took each rater about 1 h to score these responses, and they received a personalized certificate of level 3 safeguarding training if they were a working professional or a certificate of appreciation if they were a medical student.

The average of the 10 ratings was used as the response variable “NOTE.” The latter reflects the 10 raters' assessment of the extent to which the post-consultation notes suggested both an appropriate awareness of the child-safeguarding issues at stake and the development of some strategy to address those concerns. The recruitment of 10 (rather than one or two) raters reflects our awareness of the fact that this assessment is necessarily subjective and open to contestation. When dealing with such a sensitive, context-dependent situation, there is no single correct answer. Each strategy to address the possible child safeguarding concerns will not only reflect different value choices and priorities. They will also translate different assumptions under conditions of uncertainty (new patients with unknown background due to unavailability of personal notes etc.). The latter assumptions and value choices are not easily captured in a quantitative analysis, and will be the focus of a subsequent, qualitative analysis publication that takes into account the participants' post-immersion written and oral comments.

In an endeavor to collect as much pertinent data as possible, several explanatory variables were collected for analysis of covariance, given their documented impact on professional performance. These covariates were: years of experience (Dror, 2011; Dror et al., 2011), personality (Barrick and Mount, 1991), stress level (Dollard et al., 2003), and professional identification (Hekman et al., 2009). Hence, prior to the experiment, participants completed a questionnaire where they were asked whether they were a GP or a trainee GP, and how many years of experience they had since they qualified as a trainee GP. Thus our key explanatory variable -professional experience- was calculated using the GPs' year of qualification, taking into account months of career breaks. We also collected data on their personality using the 10-item NEO “big five” personality inventory (Rammstedt and John, 2007) covering Extraversion, Agreeableness, Conscientiousness, Neuroticism and Openness. GPs also completed the Perceived Stress Scale (PSS) (Cohen et al., 1983), which measures exhaustion and disengagement from work, as well as a 5-item Professional Identification Scale (PIS) (Hekman et al., 2009), which measures the extent to which individuals identify with their profession and their colleagues.

## Results

### Participants

Out of the 64 participants who attended our study, one has to be excluded due to technical issues. In total, we have 63 participants (37 GPs and 26 Trainee GPs), with age range 25-59, and 37 out of them were female. Overall, they have a mean and standard error of 10.7 ± 1.1 years of post-qualification experience as a general practitioner (see Supplementary Material: participant details).

### Note

**Figure 2:**
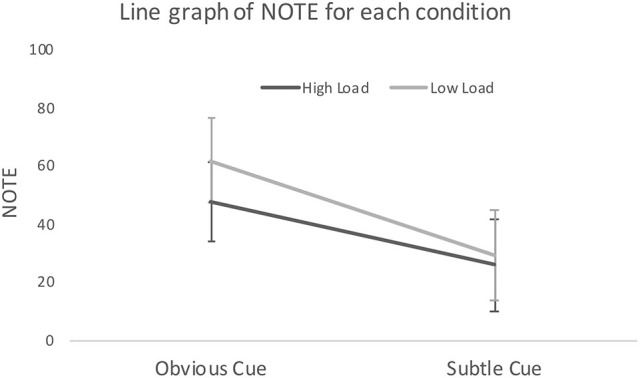
Line graph with 95% CI of NOTE, presented with different conditions.

The mean and standard error of NOTE (determined by our panel or raters, see section Response and Explanatory Variables) were 41.1 ± 4.13 (see Figure 2). A two-way ANOVA was conducted in SPSS version 24 (IBM, 2016), with the dependent variable being NOTE, independent variables being CUE and LOAD. There is no interaction effect of LOAD and CUE [*F*_(1, 62)_ = 0.53, *p* = 0.468]. CUE has a significant [*F*_(1, 62)_ = 12.68, *p* = 0.001] effect with NOTE in the obvious cue condition being higher (mean ± standard error: 54.2 ± 5.2) than NOTE in the subtle cue condition (27.6 ± 5.6), indicating that in the obvious cue condition, the notes were deemed to translate a better awareness of child-safeguarding concerns (as well as an adequate strategy to address those concerns). LOAD was not significant [*F*_(1, 62)_ = 1.35, *p* = 0.249], suggesting no difference in NOTE between the high cognitive load (mean ± standard error: 37.3 ± 5.5) and low cognitive load conditions (45.0 ± 6.1).

### Professional experience

In order to address Research Question 1 about the GP's experience and their ability to pick up child child-safeguarding concerns, we analyzed the impact of participants' professional experience on the value of NOTE. We performed two-sampled *t*-test and correlation analysis with MATLAB R2017a (MathWorks, 2018) where Pearson correlation coefficients and two-tailed *p*-values were calculated. No difference was found between GP and trainee GPs (two sample *t*-test, *p* = 0.48), neither was there a correlation between NOTE and years of experience (*R* = −0.05, *p* = 0.69). This indicates that there is no relationship between the GPs' experience and the quality of their notes. When years of experience was used as a covariate in our ANOVA analysis with SPSS, the effect of CUE (subtle vs. obvious) remains significant [*F*_(1, 62)_ = 12.304, *p* = 0.001], with no other effect found [years of experience: *F*_(1, 62)_ = 0.018, *p* = 0.893].

### Perceived stress scale (PSS) and professional identity scale (PIS)

**Figure 3:**
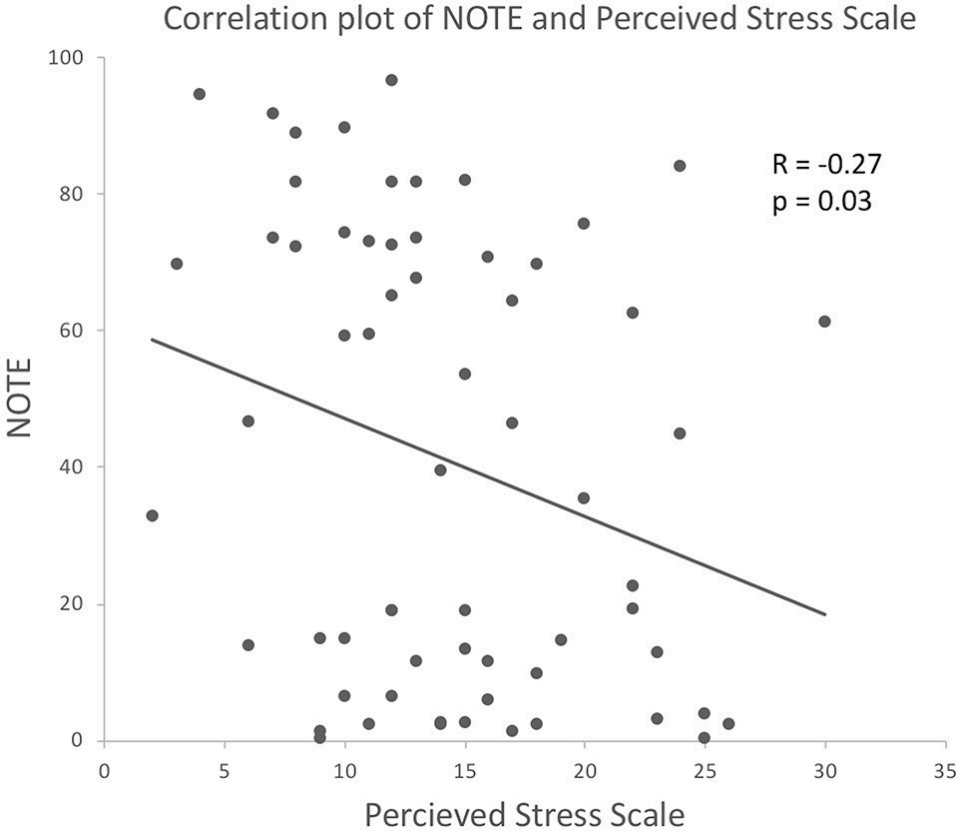
Correlation between NOTE and Perceived Stress Scale.

When PSS was used as a covariate, CUE remained significant [*F*_(1, 62)_ = 11.77, *p* = 0.001], and PSS had a significant effect [*F*_(1, 62)_ = 4.02, *p* = 0.050]. Correlation analysis of PSS and NOTE suggested a negative correlation [*R* = −0.27, *p* = 0.03], which indicates that the more stressed the participants perceived themselves to be before the experiment, the lower the quality of their notes was rated in relation to child safeguarding issues (see Figure 3).

On the other hand, PIS does not seem to explain any of the variances. When PIS was used as a covariate, CUE remain significant [*F*_(1, 62)_ = 13.82, *p* = 0.000], with no other effects found [PIS: *F*_(1, 62)_ = 2.55, *p* = 0.12].

### Personality

**Figure 4:**
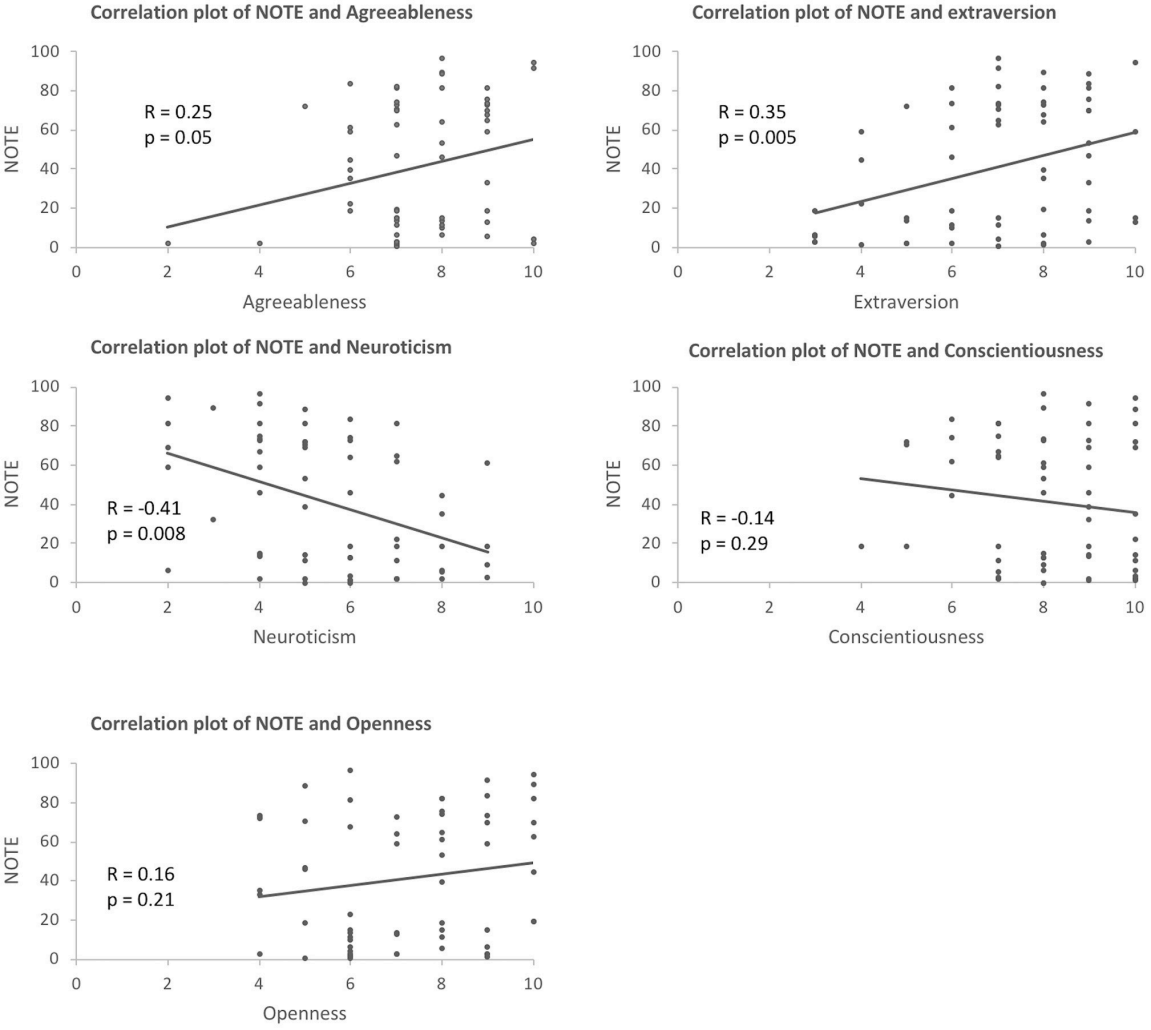
Correlation between NOTE and the five personality traits (agreeableness, extraversion, neuroticism, conscientiousness, and openness).

Amongst the NEO big-five factor personality variables, there was a significant positive correlation between NOTE and Agreeableness (*R* = 0.25, *p* = 0.05), Extraversion (*R* = 0.35, *p* = 0.005), and a negative correlation between NOTE and Neuroticism (*R* = −0.41, *p* = 0.008). No other personality traits were significantly correlated with NOTE (Conscientiousness: *R* = −0.14, *p* = 0.29; Openness: *R* = 0.16, *p* = 0.21). This suggested that those whose notes received higher marks were more agreeable, extraverted, and less neurotic (see Figure 4). Further, two-way ANOVA with each of the five personality traits as a covariate also confirmed that Agreeableness, Extraversion, and Neuroticism had an effect on NOTE [Agreeableness: *F*_(1, 62)_ = 4.75, *p* = 0.033; Extraversion: *F*_(1, 62)_ = 9.02, *p* = 0.004; Neuroticism: *F*_(1, 62)_ = 13.20, *p* = 0.001] but not Conscientiousness or Openness [Conscientiousness: *F*_(1, 62)_ = 0.29, *p* = 0.59; Openness: *F*_(1, 62)_ = 0.89, *p* = 0.35].

### Observations and comments

Our video (http://www.panxueni.com/gpcave) shows some typical interaction during our virtual consultation. It was clear from the videos that all the doctors were actively engaged in the conversation and took it seriously and reacted toward it as if it were real.

In the post-experimental questionnaire, we asked participants to comment on those aspects which contributed to the realism of their consultation experience. Among the realistic aspects, participants listed: non-verbal cues, tone of voice, patient's concerns, the commonality of the scenario (i.e., “*patients seeking support making difficult decisions*”), the responses and questions (from Chris), interaction between Chris and his son, “Him answering his phone in the middle!,” the room “*did look like a generic consulting room*.” Among the unrealistic aspects, participants listed: pauses between replies, lack of facial expressions, the inability to examine the patients, and that “*there was much less equipment than in a normal GP consultation room*.”

The participants were encouraged to leave comments about their experience—some found it stressful and challenging:

“*Impressed, evoked a sense of discomfort within me which is difficult to do in an artificial setting.”*

“*Challenging (in a positive way), fun and educational. Thanks, I'm glad I participated.”*

Many pointed out that this could be a useful tool for training, especially for medical students, here are some examples from many related comments we received:

“*It was a challenging scenario and I would have done it differently but it was interesting to watch how I acted knowing it felt wrong and I was able to reflect on it and consider how I would improve if I had to do it again”*

“*definitely has potential as a training tool, particularly with regards to difficult consultations/breaking bad news etc.”*

“*I can see how this may lower the stakes for a medical student doing consultation skills training for the first time. When I first did it I had actors and all my peers were watching on video link from the next room - it was terrifying mainly because they were real people acting very convincingly so it felt like it really mattered - plus I was being observed. This (VR) could have a role in easing med students into training in consultation skills perhaps without being observed, but as a private tool to carry out consultations and then watch yourself back and observe. Then could progress to actors with a little more confidence?”*

“*An excellent opportunity to learn/experience key scenarios and reflect/observe consultation style afterwards”*.

## Discussion

The most significant effect from our results was the safeguarding cue: those who had the less obvious cues were rated less effective in raising child protection issues in their post-consultation notes than those who had the more obvious cues. The result supports our hypothesis 2. This is encouraging as it indicates that our manipulation was successful: our virtual reality scenario and character animation were realistic in portraying the potential child abuse between the adult and child virtual characters during the consultation. It is important to note that we have manipulated only Chris' behavior and not Tom's: Chris was more violent and abusive in both his verbal and non-verbal language, however Tom behaved in exactly the same way (i.e., withdrawn in general, ducked his body when Chris got physically close, acted as if he was relieved when Chris left the room). A common recommendation in child abuse training is for practitioners to use the cues from the child rather than the adult to spot potential child abuse issues (as the adult's behavior is likely to adapt to the presence of professionals). However, our results suggest that, in practice, the adult's own behavior may play a key role in the Doctors' response.

Contrary to our expectation, our other manipulation (cognitive load) did not have a significant effect on participants' ability to notice child-safeguarding concerns, whether these participants were very experienced or not. This result contravenes Hypotheses 3 and 4: we expected GPs' situational awareness to be affected by cognitive load, and for the latter impact to be greater for less experienced GPs. Among the possible interpretations, one may point at a ceiling effect, since the cognitive load in both conditions was already relatively high. This is corroborated by the post-questionnaires results, where 56 out of 62 participants (post-questionnaire data from one participant out of the 63 was missing) reported that they found it was difficult to give advice to Chris.

This said, it is worth noting that, even if they fall some way short of significance, the load manipulation findings in the obvious cue condition are in line with hypothesis 3 and are consistent with the established literature: a higher cognitive load does impact upon GPs' ability to pick up child-safeguarding concerns. Had the effect of the difference between the two cognitive loads -high and low- been less subtle (the amplitude of this effect is difficult to determine a priori, in the absence of pilot data), our study may not have been underpowered. Future research relying on less subtle cognitive load manipulation (or greater sample size) is likely to yield significant results. If so, these results would be particularly important in terms of designing low-cost interventions aimed at improving the detection of child protection issues: it could lead to stricter guidelines when it comes to communication clarity between specialists and GPs, for instance.

As for our first research question: the participants' years of professional experience was not shown to have any effect on their ability to pick up child-safeguarding concerns. Among the possible interpretations of this result, one may point at the possibility that the skilled intuition effect (on which hypothesis 1 was based) was curbed by other factors. Among these, one may highlight the fact that less experienced GPs will have had recent and systematic training in child protection as part of their undergraduate degree (experienced GPs will have had some compulsory, continuous education training too, but the effect may not be the same). Another interesting factor that may have played a role is the desensitization that comes with repeated exposure to a particular stimulus. The possible interaction between these different factors could point at fruitful avenues for future research.

Interestingly, incidental results suggest that personal circumstances and personality traits play an important role in doctors' ability to identify child abuse issues. In particular, our results suggest that the quality of notes is negatively associated with both the participants' perceived level of stress and their level of neuroticism, while it is positively associated with their agreeableness and extraversion. In other words, those who are less stressed, less neurotic, more agreeable and extraverted tend to be better at raising the child abuse issues in their notes. It is also worth pointing out that among these traits, the effect of neurotic and extraversion is particularly strong (i.e., *p* < 0.005). One interpretation of these effects is that those who have better interpersonal skills in general experience the whole situation with Chris as less stressful and less cognitively demanding, which allows them to pay more attention to Tom.

This paper points at many potentially fruitful areas for future research. Further cognitive load manipulations in particular could prove insightful and lead to simple but high-impact public interventions, such as urging specialists to use an easy-to-read, bullet point based template for their letters to GPs. The incidental results, particularly those related to stress, would also warrant further studies aimed specifically at developing improved ways of supporting time-poor GPs who are confronted on a daily basis with emotionally charged, difficult situations. Most importantly, it is clear from the participants' comments that Immersive Virtual Reality has considerable potential as a training tool: while it is already extensively used for hands-on technical training (to master various surgery techniques for instance), its potential to train healthcare providers who are to face difficult social interactions (such as pushy patients demanding antibiotics, Pan et al., 2016) is still under-appreciated, given its advantages in terms of replicability and scalability. In the domains of mental health and pediatrics, where the use of actors can be particularly problematic, immersive virtual reality allows for a unique chance to apprehend difficult situations in a way that allows for both repetitive immersion and group discussions aimed at teasing out ethical quandaries.

This experiment also allowed to put together a wealth of qualitative data that will be analyzed in subsequent publications. Among other things, this will allow for a more fine-tuned and contextual understanding of the value choices and assumptions made by GPs under conditions of uncertainty. We will also seek to gain a better understanding of the potential which such a virtual experience may have as a continuing education tool within GPs' professional practice.

## Data availability statement

The raw data supporting the conclusions of this manuscript will be made available by the authors, without undue reservation, to any qualified researcher.

## Author contributions

XP made a substantial contribution to the conception and design of the work (involved in the initial design, wrote the script, helped with the ethics application), implementation (supervised and coordinated the implementation, directed the motion capture session, helped to get it to work in the CAVE), data acquisition (coordinated the recruitment and experiments), data analysis (conducted the data analysis and produced all the plots), and interpretation of data for the work, as well as drafting the manuscript (wrote section Results, the majority of section Materials and Methods and part of sections Introduction and Discussion). SD made a substantial contribution to the conception and design of the work (led the initial design, led the ethics application), data acquisition (coordinated the recruitment and addressed any concern from participants), interpretation of data for the work, and drafting the manuscript (wrote the majority of section Introduction and Discussion and a small part of section Materials and Methods). CF made a substantial contribution to the conception and design of the work (involved in the initial design and script writing, supervised the child abuse cues), data acquisition (supervised recruitment), interpretation of data for the work as well as drafting the manuscript (contributed to section Introduction and commented on the rest). TC-W, BC, and OD made substantial contribution to data acquisition (TC-W and BC conducted the experiment, OD was in charge of recruitment). AA, HB, and MG made substantial contribution to the conception and design of the work and implementation (HB and MG were involved in adapting the script to VR, design and implemented the user interaction; HB ran the motion capture session and implemented part of the animation; AA implemented the animation and programmed the interaction). DS made substantial contribution to the implementation and data acquisition of the work (supervised the implementation in the CAVE and technical issues during the data acquisition). PP made substantial contribution to the conception and design of the work (involved in the initial design and data analysis).

### Conflict of interest statement

The reviewer, RW, declared a shared affiliation, with no collaboration, with several of the authors, AA, BC, OD, DS, PP, CF, to the handling Editor. The remaining authors declare that the research was conducted in the absence of any commercial or financial relationships that could be construed as a potential conflict of interest.
