# Supplementary material for: A Study of Professional Awareness Using Immersive Virtual Reality: The Responses of General Practitioners to Child Safeguarding Concerns
Source: Front Robot AI. 2018 Jul 12;5:80. doi: 10.3389/frobt.2018.00080 (PMC7805796; doi:10.3389/frobt.2018.00080)
Supplement: Supplementary file 1 [file Data_Sheet_1.pdf]

frontiers

Supplementary Material

A study of professional awareness using immersive virtual reality: the responses of general practitioners to child safeguarding concerns

Xueni Pan[1], Tara Collingwoode-Williams[1], Angus Antley[2], Harry Brenton[3], Benjamin Congdon[2], Olivia Drewett[4], Marco Gillies[1], David Swapp[2], Pascoe Pleasence[5], Caroline Fertleman[6,7], Sylvie Delacroix[8]*

[1]Department of Computing, Goldsmiths, University of London, London, United Kingdom

[2]Department of Computer Science, University College London, London, United Kingdom

[3]BespokeVR Ltd, London, United Kingdom

[4]Medical School, University College London, London, United Kingdom

[5]Faculty of Laws, University College London, London, United Kingdom

[6]Great Ormond Street Institute of Child Health, University College London, London, United Kingdom

[7]Whittington Health, London, United Kingdom

[8]Birmingham Law School, University of Birmingham, Birmingham, United Kingdom

*** Correspondence:**
Sylvie Delacroix
S.Delacroix@bham.ac.uk

1 Supplementary Video – Video of the scenario

Video link: http://www.panxueni.com/gpcave

The video was filmed from on top of the Cave. Since the Cave display is stereo the images look blurred.

2 Supplementary Table – Participant details

	All (N = 63)		HO (N = 17)		HS (N = 15)		LO (N = 15)		LS (N=16)	
	Mean	SE	Mean	SE	Mean	SE	Mean	SE	Mean	SE
**Age**	35.3	1.1	34.1	1.8	34.8	2.1	33.7	1.5	38.4	2.9
**Years of experience**	10.7	1.1	9.7	1.9	11.0	2.1	9.0	1.6	12.9	2.7
**PSS**	14.3	0.8	14.0	1.4	13.6	1.5	13.3	1.7	16.1	1.6
**PIS**	5.0	0.1	5.1	0.2	4.9	0.2	5.0	0.3	5.0	0.2
**Extraversion**	7.0	0.2	7.3	0.5	6.4	0.6	6.9	0.4	7.2	0.5
**Agreeableness**	7.5	0.2	7.7	0.4	7.6	0.2	7.4	0.4	7.3	0.4
**Conscientiousness**	8.2	0.2	8.1	0.3	8.0	0.4	7.7	0.5	8.8	0.3
**Neuroticism**	5.5	0.2	5.0	0.3	5.7	0.6	5.6	0.5	5.8	0.4
**Openness**	7.2	0.2	7.1	0.4	7.3	0.5	7.7	0.5	6.8	0.5

Supplementary table 1. Participant details. Descriptive statistics of participants' age, years of experience, PSS (perceived stress scale), PIS (professional identification scale) and the big 5 personality traits. Presented as overall and also in four conditions (HO: high load, obvious cue; HS: high load, subtle cue; LO: low load, obvious cue; LS: low load, subtle cue).

3 Supplementary Data Sheet – Consultation Letters

There were two consultation letters given to the GPs prior to the virtual consultation, displayed on the laptop. They also had access to the letters during the consultation. There are four different version of the letters with different combination of recommended treatment (TAVI or Valve replacement) and how easy the letter it is to read (*easy*, *hard*).

Each doctor saw two letters, one recommending TAVI the other Valve replacement. Both letters were either *easy* or *hard* depending on the experimental condition the participant was in.

The following are the four letters, presented in the order of: TAVI *hard*, TAVI *easy*, Valve replacement *hard*, and Valve replacement *easy*.

Excellence is our moto here at UHBC

Dr Theo Gorvachio
Consultant Cardiologist
University Hospital of Barist City
Loganne Way, LEICS PG3 4ER

Dr GP Practicarr
Tree Tops GP Practice
Heaven Drive, Patrickstown, Leicestershire PG11 5NF

Christopher Truman. NHS number 1234567890. Hospital number UH243387 Age 46.
23 Trylock Close, Patrickstown, Leics PG12 8LR

Dear Dr Practicarr

Diagnoses

1. Severe asymptomatic aortic stenosis (Ejection Fraction 20%) although not impacting on his life
2. End stage renal failure on haemodialysis
3. Hypertension – on medication ?? well-controlled
4. Spinal stenosis – awaiting operative management

Many thanks for your letter regarding this 46 year old man who was found to have severe aortic stenosis during workup for spinal surgery.

His echocardiogram has demonstrated a mean gradient of 45mmHg with a calculated valve area of 0.75cm^2. The mitral valve is normal and left ventricular function remains good. He has no symptoms relating to aortic stenosis but this is likely to reflect his substantially reduced exercise tolerance as a result of spinal stenosis. I note he mobilises with a stick and has been advised to use a frame to avoid traumatic falls.

He has end stage renal failure as a result of polycystic kidney disease and is on a programme of thrice weakly haemodialysis. I understand he has been dialysed for 5 years now and it is not entirely clear to me why he has not been considered for renal transplantation

Management

There is no doubt spinal surgery would involve an elevated risk in the context of severe aortic stenosis and I would certainly recommend aortic valve intervention prior to spinal surgery. The options here are either conventional aortic valve replacement (with a biological or a mechanical valve) or transcatheter aortic valve intervention (TAVI). The risks of conventional aortic valve replacement surgery will be increased by his relative frailty – presumably due to prolonged end stage renal failure – and his inability to mobilise promptly post operatively. There are of course concerns about biological valve longevity, whether surgical or TAVI, but this may be less relevant for Mr Truman in view of his uncertain prognosis and the risks of long term anti-coagulation in this clinical context (ESRF etc). Thus, I would recommend that he undergoes a TAVI protocol CT and if this suggests a TAVI would be reasonably straightforward then I think he is likely to benefit most from this less invasive procedure. I would hope he will receive an appointment for the CT in the next few weeks and he will be discussed in the TAVI MDT shortly thereafter. I will be in touch with the consensus opinion.

Finally, he seems keen to proceed with TAVI but I understand his local cardiologist has referred him for aortic valve replacement surgery and if ultimately he changes his mind and decides to undergo surgery then I have asked him to get in touch with Eleanor Sparks (CNS) on 07784 234124 to cancel the TAVI CT.
Yours sincerely

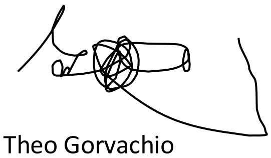

Dr Theo Gorvachio
Consultant Cardiologist. PhD. MB BChir. MA Cantab. MSc.

cc patient

Excellence is our moto here at UHBC

Dr Theo Gorvachio
Consultant Cardiologist
University Hospital of Barist City
Loganne Way, LEICS PG3 4ER

Dr GP Practicarr
Tree Tops GP Practice
Heaven Drive
Patrickstown
Leicestershire PG11 5NF

Christopher Truman. NHS number 1234567890. Hospital number UH243387 Age 46.
23 Trylock Close, Patrickstown, Leics PG12 8LR

Dear Dr Practicarr

Diagnoses

1. Severe asymptomatic aortic stenosis (Ejection Fraction 20%) although not impacting on his life
2. End stage renal failure on haemodialysis
3. Hypertension
4. Spinal stenosis – awaiting operative management

Opinion

In view of morbidity and frailty would recommend TAVI as lower operative risk for this procedure.

Yours sincerely

Dr Theo Gorvachio
Consultant Cardiologist. PhD. MB BChir. MA Cantab. MSc.

cc patient

Dr Matthew Valve
Consultant Cardiologist
St Edmund's General Hospital
Golding Road
Patrickstown
PG14 3NG

Local NHS serving the local people

Dr GP Practicarr
Tree Tops GP Practice
Heaven Drive
Patrickstown
Leicestershire PG11 5NF

Christopher Truman. NHS number 1234567890. Hospital number SE13247 Age 46.
23 Trylock Close, Patrickstown, Leics PG12 8LR

Dear Gerrard

Thank you for referring Mr Chris Truman who is a delightful 46 year old gentleman with an extensive past medical history. As you well know he has been on thrice weekly haemodialysis via a cimino fistula in his left forearm. He has end stage renal failure secondary to poorly controlled hypertensive disease in combination with bilateral polycystic kidney disease. He goes to St Evelina's on a Monday, Wednesday and Friday afternoon using the hospital transport service. As you are aware he has limited mobility because of his increasingly severe spinal stenosis. He is using a single wooden walking stick although I understand from him you have suggested he uses a more sophisticated walking aide with wheels or one like a zimmer frame. I understand he was being worked up for spinal surgery and the anaesthetist picked up an ejection systolic murmur radiating to the carotids consistent with aortic stenosis. On examining him today I would agree with this finding and would also comment he has a slow I rising pulse. Consistent with this, an echocardiogram has demonstrated severe aortic stenosis with a peak gradient of 85mmHg. Fortunately left ventricular function remains good. He has no symptoms relating to the aortic stenosis, specifically he has no problems with dyspnoea or chest pain and has never been syncopal.

Severe aortic stenosis would certainly increase the risks of spinal surgery and I did discuss with him how keen he was to proceed with the operation. He, however, is clear that his quality of life is atrocious at the moment with persistent back pain and marked limitation of mobility. I would thus suggest we consider referring him on for conventional aortic valve replacemement surgery prior to him undergoing spinal surgery. I guess a biological valve could be considered but at his age I would imagine he would benefit most from mechanical aortic valve replacement. Needless to say the ultimate decision about the type of valve used will be left to Mr Truman to discuss with the cardiac surgeon.

I have taken the liberty of referring him onto Mr John Smith, cardiac surgeon at Thomas' University Hospital. They have opened up a number of Saturday lists as part of a waiting list initiative and he would be ideal to benefit from one of these as he can have his dialysis the day before as per his normal routine. I have no doubt we will be hearing from John in the near future.

With kind regards

Dr Emen VandErg MD MBBS FRCP

cc Mr Truman

Dr Matthew Valve
Consultant Cardiologist
St Edmund's General Hospital
Golding Road
Patrickstown
PG14 3NG

Local NHS serving the local people

Dr GP Practicarr
Tree Tops GP Practice
Heaven Drive
Patrickstown
Leicestershire PG11 5NF

Christopher Truman. NHS number 1234567890. Hospital number SE13247 Age 46.
23 Trylock Close, Patrickstown, Leics PG12 8LR

Dear Gerrard

Diagnoses

1. Aortic stenosis
2. CKD stage 5 – on dialysis
3. High blood pressure
4. Spinal stenosis

Opinion

Mechanical aortic valve replacement is preferred in view of his young age.

Management

I have referred him to the cardiothoracic surgeons to list him

With kind regards

Dr Emen VandErg MD MBBS FRCP

cc Mr Truman
